# A dynamic causal model study of neuronal population dynamics

**DOI:** 10.1016/j.neuroimage.2010.01.098

**Published:** 2010-05-15

**Authors:** André C. Marreiros, Stefan J. Kiebel, Karl J. Friston

**Affiliations:** The Wellcome Trust Centre for Neuroimaging, Institute of Neurology, University College London, UCL, 12 Queen Square, London, UK WC1N 3BG, UK

**Keywords:** Neural-mass models, Nonlinear, Modelling, Laplace assumption, Mean-field, Neuronal, Bayesian

## Abstract

In this paper, we compare mean-field and neural-mass models of electrophysiological responses using Bayesian model comparison. In previous work, we presented a mean-field model of neuronal dynamics as observed with magnetoencephalography and electroencephalography. Unlike neural-mass models, which consider only the mean activity of neuronal populations, mean-field models track the distribution (e.g., mean and dispersion) of population activity. This can be important if the mean affects the dispersion or *vice versa*. Here, we introduce a dynamical causal model based on mean-field (i.e., population density) models of neuronal activity, and use it to assess the evidence for a coupling between the mean and dispersion of hidden neuronal states using observed electromagnetic responses. We used Bayesian model comparison to compare homologous mean-field and neural-mass models, asking whether empirical responses support a role for population variance in shaping neuronal dynamics. We used the mismatch negativity (MMN) and somatosensory evoked potentials (SEP) as representative neuronal responses in physiological and non-physiological paradigms respectively. Our main conclusion was that although neural-mass models may be sufficient for cognitive paradigms, there is clear evidence for an effect of dispersion at the high levels of depolarization evoked in SEP paradigms. This suggests that (i) the dispersion of neuronal states within populations generating evoked brain signals can be manifest in observed brain signals and that (ii) the evidence for their effects can be accessed with dynamic causal model comparison.

## Introduction

Neuronal activity generated by coupled neuronal populations can be autonomous or elicited by sensorimotor or cognitive perturbation. Neural models have been in used to study this activity for many years (e.g., Wilson and Cowan 1972; Nunez 1974; Freeman 1975; Lopes da Silva et al., 1976; Jansen and Rit, 1995; Jirsa and Haken 1996; Valdes et al., 1999; David and Friston 2003). These models form the basis for understanding the genesis of neuroimaging signals (e.g., Riera et al., 2007). We continue this modelling initiative, by using the formulation of population dynamics described by Marreiros et al. (2009) as the basis of a dynamic causal model (DCM) of evoked electromagnetic responses. We try to establish its face validity using Bayesian model comparison and evaluate its ability to explain empirical data, in relation to simpler variants.

This is the third and final paper in a trilogy that tries to integrate the Fokker-Planck formalism and stochastic formulations of neuronal dynamics with DCM. The first (Marreiros et al. 2008) introduced the idea that the sigmoid activation could be regarded as accommodating dispersion of neuronal states (as opposed to dispersion of the threshold). The second (Marreiros et al. 2009) used this perspective to motivate a simple method-of-moments scheme to model density dynamics in terms of the mean and dispersion of neuronal states. This simplicity is important because the current paper uses this scheme in dynamic casual models of real data.

This work rests on a key distinction between models that summarize the activity of a neuronal population with a single state (e.g., its mean activity) and those that model the distribution of states in terms of probability densities (i.e., density dynamics). We follow the terminology established in Deco et al. (2008), which adopts a pragmatic approach: if the model can be cast as a set of ordinary differential equations describing the evolution of neuronal states, it is called a neural-mass model (NMM; e.g., Freeman 1975; Lopes da Silva et al., 1976; Rodrigues et al., 2006). This is motivated by treating the current state as a point ‘mass’ (i.e., delta function) approximation to the underlying density on the population's states. Conversely, models based on stochastic differential equations that include random fluctuations are referred to as mean-field models (MFM; e.g., Knight 1972a,b; Sampolinsky and Zippelius, 1982; Nykamp and Tranchina 2000; Omurtag et al. 2000; Frank et al., 2001; Haskell et al., 2001). This nomenclature appeals to the use of the term ‘mean-field’ in statistical physics and machine learning. A mean-field assumption approximates a full density (on the states of a population of neurons or multiple populations) with a density that factorises into a series of simpler marginal densities; where the marginals influence each other through average or mean-field effects. These models necessary entail a stochastic or probabilistic representation of activity, which is usually encoded by the sufficient statistics of a probability density (like the mean and variance of a Gaussian distribution). Although we will not deal with them in this paper, models that are formulated as partial differential equations of space and time are referred to as neural field models (NFM; e.g., Wilson and Cowan 1972; Amari 1972; 1975; Robinson et al., 2005), because they model the spatiotemporal dynamics on spatially extensive (e.g., cortical) fields.

It should be noted that this operational taxonomy of models is insensitive to the deep history of their development or their original descriptions; for example, there is a fundamental difference between the work of Wilson and Cowan (1972) and that of Amari (1972; 1975) on ‘neural fields’ and that of Freeman (1975) and that of Lopes da Silva et al. (1976) on ‘neural-masses’: the former provides spatiotemporal descriptions of neuronal dynamics, without recourse to experimental evidence; whereas the latter are derived from observing empirical population responses and writing down equations that reproduce physiological responses. Both neural field and mass models are parsimonious models of mean activity (e.g., firing rate or membrane potential) and have been used to emulate a wide range of brain rhythms and dynamics. Neural-mass models are particularly appropriate for data that reflect the average behaviour of neuronal populations; such as the electroencephalogram (EEG) and magnetoencephalogram (MEG) (David et al., 2006; Marreiros et al., 2009).

In a previous paper (Marreiros et al., 2009) we formulated neural-mass models, currently used as generative models in dynamic causal modelling, as a limiting case of mean-field models; in which the variance of the activity in any one neuronal population is fixed. Unlike neural-mass models, mean-field models consider the full density on the hidden states of neuronal populations including the variance or dispersion. We derived a generic mean-field treatment of neuronal populations or ensembles based on a Laplace or Gaussian approximation to the population or ensemble density. The Laplace approximation (also known as a saddle-point approximation) approximates the integral of an exponential function using a second-order Taylor expansion. When the function is a probability density, the implicit assumption is that the density is approximately Gaussian. Because a Gaussian density can be specified in terms of its first two moments, the ensuing scheme is formally identical to the second-moment method described by Rodriguez and Tuckwell (1996). This scheme summarizes density dynamics with (ordinary differential) equations of motion for the sufficient statistics of the ensemble density. This reduces to a neural-mass model when the second-order statistics (*i.e*., variance) of neuronal states is assumed to be constant. The key behaviour we were interested in was the coupling between the mean and variance under the Laplace approximation, which is lost in neural-mass approximations. In this work, we use the mean-field density dynamics as the basis of a dynamic causal model (DCM) of observed data. The resulting framework allows one to adjudicate between models which include (or not) the high-order statistics of hidden neuronal states when predicting EEG/MEG time series. This adjudication is based on the relative evidence for different models of the same data that obtain from Bayesian inversion (i.e., fitting) of the models.

The aim of this work was to evaluate dynamic causal models based on density dynamics and compare them to established neural-mass models. DCM is a natural extension of the convolution models used in the standard analysis of biological time series (see David et al., 2006). DCM models neuronal dynamics in each source or region and interactions within and between distributed sources. Currently, DCM uses neural-mass models and implicitly considers only the mean neuronal state for each population; i.e., special cases of the more general population density formulation, in which we ignore all but the first-order statistics (i.e., the mean). Here, we replace the neural-mass model with second-order dynamics under the Laplace approximation using the Fokker-Planck formalism (Frank, 2004) to give a mean-field model (Marreiros et al., 2009). This allows us to model the interactions between mean neuronal states (e.g., firing rates) and their dispersion within each population and to compare homologous DCMs based on neural-mass and mean-field models in terms of their model evidence. This enabled us to ask whether including density dynamics is warranted; i.e., to see if there is any evidence for an effect of second-order statistics or dispersion.

The paper is composed of two sections. In the first, we summarize density dynamics under mean-field and Laplace assumptions and describe the resulting DCM framework. In the second section, we use two EEG data sets and Bayesian model comparison (BMC) to assess the relative evidence for neural-mass and mean-field models. In addition, we establish the face validity of neural-mass DCMs and their mean-field generalizations using synthetic data, generated using the conditional estimates of the network parameters, for each of the empirical examples.

## Theory

Neural-mass and field models can reproduce neuronal dynamics reminiscent of observed evoked responses. However, to emulate more complex dynamics we may need to take into account the high-order statistics of ensemble dynamics. In a previous paper (Marreiros et al., 2009) we derived a generic mean-field treatment of neuronal dynamics, based on a Laplace approximation to the ensemble density. This model is formulated in terms of equations of motion for the moments of the ensemble density, reducing to an NMM when the second-order moment (variance) is ignored. The interesting behaviour in these mean-field models arises from the coupling between the mean and variance of ensemble activity, which is ignored in neural-mass approximations. Here, we will use the Laplace and neural-mass approximations in DCMs of electrophysiological responses to sensory input. We start by reviewing briefly mean-field and neural-mass models (MFM and NMM) for M/EEG and then turn to Bayesian estimation, inference and model comparison in the context of DCM.

### Modelling neuronal dynamics with mean-field models

In DCM, neural-mass models are used to model the evolution of the mean response of neuronal populations to exogenous or experimental perturbations (David et al., 2006; Kiebel et al., 2008). Mean-field approximations go further and model the distribution of the population response. However, employing MFMs can be computationally expensive, because one has to consider the density at all points in neuronal state-space, as opposed to a single quantity (e.g., the mean). In other words, to represent the full probability distribution of states over a population, we would need to encode the probability of finding a neuron in every state at each point in time, which would require an infinite number of variables. Under the Laplace approximation, the population or ensemble density reduces to a Gaussian form, whose sufficient statistics is composed of only the conditional mean and covariance. This simplification allows one to model interactions between the first two moments (i.e., mean and variance) of neuronal states in an efficient and tractable way.

In brief, the dynamics of neurons in an ensemble can be described with the stochastic differential equation *dx* = *f*(*x,u*)*dt* + *ωd*Γ, where *f*(*x,u*) corresponds to the equations of motion describing neuronal processes and *ω* controls the amplitude of uncorrelated random fluctuations *d*Γ (i.e., a Weiner process) in neuronal states *x*(*t*)∈ℜ^*n*^. Here, *u*(*t*)∈ℜ^*m*^ is a real valued vector of time-dependent exogenous or experimental inputs. To compute the associated density dynamics, one can use the Fokker-Planck formalism: ˙*q* = −▿·*fq* *+* ▿·*D*▿*q*, where *q*(*x,t*) is an approximate density on neuronal states (e.g., post synaptic potentials and conductances) and *D*(*ω*) is a diffusion tensor. The time-dependent density *q*(*x,t*) is an ensemble density, which approximates the probability of finding a neuron from the ensemble in state *x* and time *t*. The high dimensionality and complexity of this Fokker-Planck formalism can be finessed with a mean-field approximation, *q*(*x,t*) ≈ Π *q*(*x*^(*i*)^,*t*), which describes the evolution of separable ensembles that are coupled by mean-field effects. Moreover, the Laplace assumption allows us to summarize the ensemble density with a Gaussian density, described by the mean and variance of the states of the *i*-th population. By parameterizing the densities in terms of these time-dependent sufficient statistics, we have; *q*(*x*^(*i*)^,*t*) = *N*(*μ*^(*i*)^,Σ^(*i*)^). Please see Marreiros et al. (2009) for a fuller explanation.

Crucially, the Laplace assumption allows the density dynamics (based on stochastic differential equations) to be expressed as an ordinary differential equation for its sufficient statistics (mean and variance) in a compact form (Marreiros et al., 2009)

(1) $$\begin{array}{c} {\dot{\mu }}_{j}^{\left(i\right)}=f_{j}^{\left(i\right)}(\mu ,u)+\frac{1}{2}tr(\Sigma^{\left(i\right)}\partial_{x}^{2}f_{j}^{\left(i\right)})\\ {\dot{\Sigma }}^{\left(i\right)}=\partial_{x}f^{\left(i\right)}\Sigma^{\left(i\right)}+\Sigma^{\left(i\right)}\partial_{x}f^{(i)T}+D^{\left(i\right)}+D^{(i)T} \end{array}$$

This allows one to describe the population dynamics knowing only the flow, its gradient and curvature at each point in state-space. The reason that there are no superscripts on the sufficient statistics *μ*:= {*μ*^(1)^, *μ*^(2)^,…,Σ^(1)^,Σ^(2)^,…,} is that the sufficient statistics of one population can influence the sufficient statistics of another through extrinsic and intrinsic connections. Eq. (1) shows explicitly how the first and second moments of the density depend on each other; the variance affects the mean when the curvature of the flow is nonzero (which is always the case when the equations of motion are nonlinear in the states). The effect of the mean on the variance depends on the gradients of the flow, which only change with the mean, when the curvature is nonzero. Therefore, the MFM formulation enables us to model interactions between the mean of neuronal states (e.g., postsynaptic potentials) and their variance over each population modelled (cf., Harrison et al., 2005).

A special case of the MFM is obtained if we assume Σ^(*i*)^ is fixed for all populations. This is the NMM, where we ignore all but the first moment of the density (*i.e*., the mean or mode). Because the curvature ∂_*x*_^2^*f*_*j*_^(*i*)^ is generally constant and negative, this induces a decay term, giving density dynamics of the form

(2) $${\dot{\mu }}_{j}^{\left(i\right)}=f_{j}^{\left(i\right)}(\mu ,u)+\frac{1}{2}tr(\Sigma^{\left(i\right)}\partial_{x}^{2}f_{j}^{\left(i\right)})$$

This corresponds to a neural-mass model and will be the NMM used in this paper. The value for Σ^(*i*)^ we used was the stationary solution to Eq. (1), in the absence of input (i.e., the same steady-state value as in the MFM). Further assuming that Σ^(*i*)^ is spherical (i.e., all off-diagonal terms are zero) means the decay terms disappears because the leading diagonal; ∂_*x*_^2^*f*_*j*_^(*i*)^ is generally 0 for most neuronal models. This is because the self-decay terms are generally linear in the states (see Eq. (3) below for an example). In this case, we can ignore the second-order statistics completely and the dynamics reduce to the original equations of motion for a single neuron: ˙*μ*^(*i*)^ = *f*^(*i*)^(*μ,u*).

The equations of motion *f*^(*i*)^ for each population considered in this paper are described in Marreiros et al. (2009) and conform to a simplified Morris and Lecar (1981) model, where the states are composed of transmembrane potential and three conductances: leaky, excitatory and inhibitory. This conductance-based model can be expressed with the equations of motion for the states of the *i*-th population

(3) $$\begin{array}{c} x^{\left(i\right)}=\left[\begin{array}{c} V^{\left(i\right)}\\ g_{E}^{\left(i\right)}\\ g_{I}^{\left(i\right)} \end{array}\right]\\ f^{\left(i\right)}=\left[\begin{array}{c} \frac{1}{C}(g_{L}(V_{L}-V^{\left(i\right)})+g_{E}^{\left(i\right)}(V_{E}-V^{\left(i\right)})+g_{I}^{\left(i\right)}(V_{I}-V^{\left(i\right)})+\gamma_{i}^{u}u)\\ \kappa_{E}(\varsigma_{E}^{\left(i\right)}-g_{E}^{\left(i\right)})\\ \kappa_{I}(\varsigma_{I}^{\left(i\right)}-g_{I}^{\left(i\right)}) \end{array}\right]\\ \varsigma_{k}^{\left(i\right)}=\sum_{j}\gamma_{ij}^{k}\sigma (\mu_{V}^{\left(j\right)}-V_{R},\Sigma^{\left(j\right)}) \end{array}$$

The presynaptic input *ς*_*k*_^(*i*)^:*k*∈*E*,*I* scales with the expected firing rate in all populations, where the sigmoid function *σ*(·) is a Gaussian cumulative density on the depolarization (Marreiros et al., 2008) and the coupling parameters *γ*_*ij*_^*k*^ specify the connectivity among populations. *V*_*R*_ is the threshold potential for neuronal firing. These parameters can be used to ensure that each population couples to one and only one conductance type (*i.e*., each population can only release one sort of neurotransmitter). Critically, in this work, the model also allows for conduction delays on the connections (not shown in the equations for simplicity). Given Eq. (3) we can write down the derivatives that are required to evaluate the density dynamics in Eq. (1) (cf. Eq. (18) in Marreiros et al. 2009), where, dropping superscripts for clarity:

(4) $$\begin{array}{c} \partial_{x}f=\left[\begin{array}{ccc} -\frac{1}{C}\sum_{k}g_{k} & \frac{1}{C}(V_{E}-V) & \frac{1}{C}(V_{I}-V)\\ 0 & -\kappa_{E} & 0\\ 0 & 0 & -\kappa_{I} \end{array}\right]\\ \partial_{x}^{2}f_{V}=\left[\begin{array}{ccc} 0 & -\frac{1}{C} & -\frac{1}{C}\\ -\frac{1}{C} & 0 & 0\\ -\frac{1}{C} & 0 & 0 \end{array}\right]\\ \partial_{x}^{2}f_{g}=\left[\begin{array}{ccc} 0 & 0 & 0\\ 0 & 0 & 0\\ 0 & 0 & 0 \end{array}\right] \end{array}$$

This particular form of neuronal model is among the simplest that are nonlinear in the states (note that the rate of change of voltage depends on conductance times voltage). This nonlinearity is critical in the present context because, as discussed above, in its absence there is no coupling between the mean and dispersion (i.e., the neural-mass and mean-field formulations would behave identically). We are not suggesting, in the choice of this model, that it is a sufficient or complete model of neuronal dynamics; this would be a wider question for model comparison. We are using this minimal model to ask whether mean-field formulations provide a better account of observed neuronal responses than their neural-mass counterparts.

Having established the form of the ordinary differential equations (Eqs. (1) and (2)) for the mean-field and neural-mass models of population activity, we now describe how they are embedded in a DCM to provide a spatiotemporal forward model of observed electromagnetic responses.

### Dynamic causal modelling for EEG/MEG

Dynamic causal modelling provides a generative model for M/EEG responses (David et al., 2006; Kiebel et al., 2008). The idea behind this approach is that M/EEG data are the response of a distributed network of interacting neuronal sources to experimental inputs. Here every source contains different neuronal populations, each described by a NMM or a MFM. Each population has its own (intrinsic) dynamics governed by the neural-mass or the mean-field equations above, but also receives extrinsic input, either directly as sensory input or from other sources. The dynamics of these sources are specified fully by a set of first-order differential equations that are formally related to other neural-mass and mean-field models of M/EEG (e.g., Breakspear et al. 2006; Rodrigues et al. 2006).

**Fig. 1:**
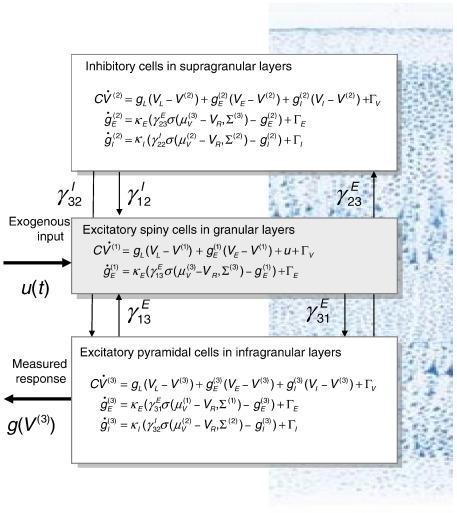
Neuronal state-equations for a source model with a layered architecture composed of three interconnected populations (spiny-stellate, interneurons, and pyramidal cells), each of which has three different states (voltage, excitatory and inhibitory conductances).

DCM for event related potential models the activity of a source using three neural subpopulations, each assigned to one of three cortical layers; an excitatory subpopulation in the granular layer, an inhibitory subpopulation in the supra-granular layer and a population of deep pyramidal cells in the infra-granular layer. These are connected using the connectivity rules described in the study of Felleman and Van Essen (1991). See Fig. 1. Here, it is assumed that the depolarization of pyramidal cell populations gives rise to observed M/EEG data, which are expressed in the sensors through a conventional lead-field. The full spatiotemporal model takes the form of a nonlinear state-space model with hidden states modelling (unobserved) neuronal dynamics, while the observation (lead-field) equation is instantaneous and linear in the states. The ensuing DCM is specified in terms of its state-equation (Eqs. (1) or (2)) and an observer or output equation

(5) $$h(\vartheta )=L(\theta )\left[\begin{array}{c} \alpha_{1}\mu_{V}^{\left(1\right)}\\ \alpha_{2}\mu_{V}^{\left(2\right)}\\ \vdots \end{array}\right]$$

where *μ*_*V*_^(*i*)^ is the mean depolarization of the *i*-th population and *h*(*θ*) is the predicted EEG or MEG signal. Here, *ϑ*⊃{*θ*,*α_i_*,*γ_ij_^k^*,*κ*_*I*_,*κ*_*E*_,*ω*…) are the unknown quantities that parameterize the state and observer equations (David et al., 2006). The parameters also control any unknown attributes of the stimulus function encoding exogenous input; we use Gaussian bump functions parameterized by their latencies and dispersions. We assume the MEG or EEG signal is a linear mixture of depolarizations in the pyramidal populations; where the columns of *L*(*θ*) are conventional lead-fields, which account for passive conduction of the electromagnetic field from the sources to the sensors (Mosher et al. 1999). The parameters of the lead-field, *θ* encode the location and orientation of the underlying sources and *α* scale their contribution to the dipole. Please see David et al. (2006) and Kiebel et al. (2008) for further background and Daunizeau et al. (2009) for details of the particular spatial model we used in this study.

The predicted signal *h*(*θ*) corresponds to a generalized convolution of exogenous inputs (i.e., experimental stimulus functions). Under Gaussian assumptions about measurement noise, this generalized convolution gives a likelihood model for observed M/EEG data:

(6) $$\begin{array}{c} y=\text{vec}(h(\vartheta )+X\beta )+ɛ\Rightarrow \\ p\left(y|\vartheta ,\lambda \right)=N(\text{vec}(h(\vartheta )+X\beta ),\text{diag}(\lambda )⊗V) \end{array}$$

Observation noise, *ɛ*, is assumed to be zero-mean Gaussian and independent over channels, where *λ* is a vector of unknown channel-specific error variances and *V* represents a temporal autocorrelation matrix. Low-frequency noise or drift components are modelled by confounding variables in the columns of the matrix, *X* (this was simply a constant term in this paper) with associated parameters β ⊂ *ϑ*. For computational expediency, we reduce the dimensionality of the sensor data, while retaining the maximum amount of information. This is assured by projecting the data onto a subspace defined by its principal modes; computed using singular value decomposition; see Fastenrath et al. (2009). Having established how the mean-field and neural-mass models specify a likelihood model for observed signals, we now consider how the ensuing DCM is fitted or inverted.

### Bayesian estimation, conditional inference and model comparison

A DCM is fitted to data by tuning the free parameters to minimize the discrepancy between predicted and observed MEG/EEG time series, under complexity constraints. In addition to minimizing prediction error, the parameters are constrained by a prior specification of the range they are likely to lie in the study of Friston et al. (2003). These constraints, which take the form of a prior density −*p*(*ϑ*), are combined with the likelihood, *p*(*y*|*ϑ*), to form a posterior density *p*(*ϑ*|*y*) ∝ *p*(*y*|*ϑ*)*p*(*ϑ*) according to Bayes' rule. The priors *p*(*ϑ*) are usually specified under log-normal assumptions to impose positivity constraints; and are therefore specified by the prior mean and variance of log-parameters. Table 1 lists the priors for the free parameters of the neuronal model and the values we used for its fixed parameters.

For a given DCM, say model *m*, inversion corresponds to approximating the moments of the posterior or conditional distribution given by Bayes' rule

(7) $$p\left(\vartheta |y,m\right)=\frac{p\left(y|\vartheta ,m\right)p\left(\vartheta ,m\right)}{p\left(y|m\right)}$$

The estimation procedure employed in DCM is described in the study of Friston et al. (2003). The posterior moments (mean and covariance) are updated iteratively using variational Bayes under a fixed-form Laplace (i.e., Gaussian) approximation to the conditional density −*q*(*ϑ*). This can be regarded as an expectation-maximization (EM) algorithm that employs a local linear approximation of the predicted responses (Eq. (6)) about the current conditional expectation. The E-step conforms to a Fisher-scoring scheme (Fahrmeir and Tutz 1994) that performs a descent on a variational free-energy *F*(*q*,*λ*,*m*), with respect to the conditional moments. In the M-step, the error variances *λ* are updated in exactly the same way to provide their maximum likelihood. The estimation scheme can be summarized as follows:

(8) $$\begin{array}{c} E\text{−step}:q\leftarrow \underset{q}{\min}F(q,\lambda ,m)\\ M\text{−step}:\lambda \leftarrow \underset{\lambda }{\min}F(q,\lambda ,m)\\ F(q,\lambda ,m)={\langle \ln q(\vartheta )-\ln p(y|\vartheta ,\lambda )-\ln p(\vartheta |m)\rangle }_{q}\\ =KL(q\|p(\vartheta |y,\lambda ))-\ln p(y|\lambda ,m) \end{array}$$

The free-energy is simply a function of the log-likelihood, the log-prior and the approximation to the conditional density we seek. The free-energy is the Kullback-Leibler divergence between the real and approximate conditional density minus the log-likelihood. This means that when the free-energy is minimized, the discrepancy between the true and approximate conditional density is suppressed (because the divergence is non-negative). At this point the free-energy approximates the negative log-evidence *F* ≈ −ln *p*(*y*|*λ*,*m*). This scheme is identical to that employed by DCM for fMRI and evoked responses (Friston et al., 2003; David et al., 2006).

The log-evidence is an important quantity because it allows one to compare different models (Penny et al. 2004). We can approximate the log-evidence for model *m* with ln *p*(*y*|*m*) ≈ −*F*. The most likely model is the one with the largest log-evidence. Model comparison rests on the likelihood ratio (i.e., Bayes-Factor) of the evidence or relative log-evidence for two models. For models *i* and *j* the Bayes-Factor is

(9) $$\begin{array}{ccc} B_{ij}=\frac{p\left(y|m_{i}\right)}{p\left(y|m_{j}\right)} & \Rightarrow & \ln B_{ij}=\ln p\left(y|m=i\right)-\ln p\left(y|m=j\right) \end{array}$$

Strong evidence in favour of one model typically requires the difference in log-evidence to be three or more (Penny et al. 2004). Under flat priors on models this corresponds to a conditional confidence that the winning model is exp(3) ≈ 20 times more likely than the alternative. This indicates that the data provide ‘strong’ (10:1 to 30:1) evidence in favour of one model over the other. See http://en.wikipedia.org/wiki/Bayes_factor for the range of Bayes factors indicating ‘very strong’ (30:1 to 100:1) and ‘decisive’ (more than 100:1) evidence for a model. In the next section, we will use the free-energy bound on log-evidence to compare the different models elaborated above.

## Simulations and empirical results

Our key question was: can we find evidence for coupling between the mean and dispersion of neuronal states in empirical data? However, we anticipated that the answer would be context sensitive; in the sense that some evoked responses may induce large fluctuations in dispersion, whereas others may not. This context sensitivity can be seen from the form of Eq. (1), where changes in the dispersion of neuronal states depend upon the systems Jacobian ∂*_x_ f*^(*i*)^ or rate of change of flow with state. The Jacobian depends on depolarization and conductance (Eq. (4)), which depends on presynaptic input *ς*_*k*_^(*i*)^. This implies that we would expect to see large fluctuations in dispersion and the ensuing effect on the mean under high levels of extrinsic presynaptic input. We therefore chose to perform our model comparison using two sorts of evoked responses. The first used a traditional ‘cognitive’ paradigm (a mismatch negativity paradigm) in which auditory stimuli can be regarded as delivering low amplitude physiological inputs to cortical sources. In contrast, the second paradigm was a somatosensory evoked potential (SEP) paradigm; in which neuronal sources are excited with a non-physiological electrical stimulus, eliciting transient but high amplitude presynaptic inputs. We predicted that if there was any evidence for the mean-field model, relative to the neural-mass model, then we would be more likely to see it in the SEP paradigm, relative to the mismatch negativity paradigm. In what follows, we describe these paradigms and the results of our model comparisons.

### Mismatch negativity paradigm

In this section, we analyze data from a multi-subject mismatch negativity (MMN) study (Garrido et al. 2007a). This analysis is largely the same as we have presented previously when looking at plasticity using DCM. The only difference here is that we used the conductance-based neuronal model described in Marreiros et al. (2009) and compared the neural-mass and mean-field variants of this model. In brief, the MMN is the differential response to an unexpected (rare or oddball) auditory stimulus relative to an expected (standard) stimulus. The MMN has been studied extensively and is regarded as a marker for error detection, caused by a deviation from a learned regularity, or familiar auditory context. According to Näätänen et al. (2001) the MMN is caused by two underlying functional processes, a sensory memory mechanism and an automatic attention-switching process that might engage frontal generators (Giard et al. 1990). It has been shown that the temporal and frontal MMN sources have distinct behaviours over time (Rinne et al. 2000) and that these sources interact with each other (Jemel et al. 2002). Thus the MMN could be generated by a temporofrontal network (Doeller et al. 2003; Opitz et al. 2002; Escera et al. 2003), as revealed by M/EEG and fMRI studies. In a predictive coding framework, these findings can also be framed as adaptation and experience-dependent plasticity in an auditory network (Jääskeläinen et al., 2004; Friston, 2005; Garrido et al., 2007a,b).

**Fig. 2:**
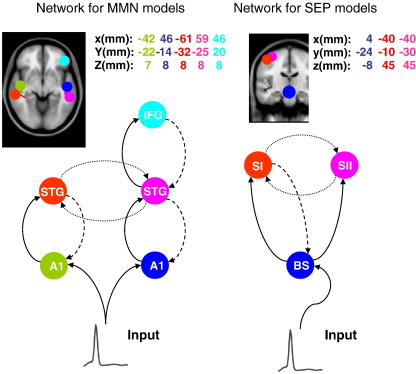
DCM networks used for the mismatch negativity (MMN) and somatosensory evoked potential (SEP) paradigms. Forward connections (solid lines), backward connections (dashed lines) and lateral connections (dotted lines) couple sources. The inserts shows the prior locations of sources; these are composed of vertices on a canonical cortical mesh within 16 mm of the source locations shown in the Talairach and Tournoux coordinates. See text and Garrido et al. (2007a) for a full model description.

Using DCM, we modelled the MMN generators with a temporofrontal network composed of bilateral sources over the primary and secondary auditory and frontal cortex. Following Garrido et al. (2007a), we used a five-source network with forward and backward extrinsic (between-source) connections. Exogenous or auditory input (modelled with *u*(*t*)∈ℜ, a parameterized bump function of time; see Table 1) enters via subcortical structures into two bilateral sources in posterior auditory cortex (lA1 and rA1). These have forward connections to two bilateral sources in anterior auditory cortex; i.e., superior temporal gyri (lSTG and rSTG). These sources are laterally and reciprocally connected via the corpus callosum. The fifth source is located in the right inferior frontal gyrus (rIFG) and is connected to the rSTG with reciprocal unilateral connections. Using these sources and prior knowledge about the functional anatomy cited above, we specified the DCM network in Fig. 2. Here, we were interested in comparing the NMM and MFM formulations of this network, in terms of their negative free-energy. To simplify the analysis, we modelled only the responses evoked by standard stimuli (from 0 ms to 256 ms).

### Empirical results

**Fig. 3:**
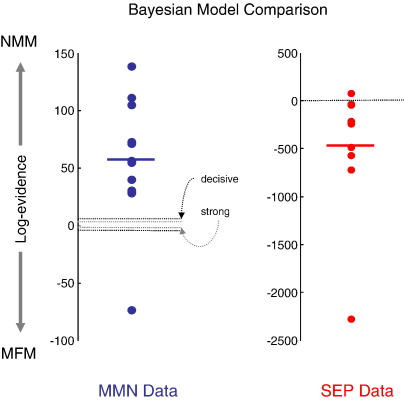
Bayesian model comparisons for NMM in relation to MFM. Right: relative log-evidence for the NMM for each subject using the network in Fig. 2. The NMM log-evidences are consistently better than the MFM log-evidences, with a pooled difference > 100 over subjects. Left: the same results for the SEP data; the group log-evidence difference was > 100 in favour of the MFM. The solid lines indicate the mean over subjects.

Two DCMs (NMM and MFM variants) were inverted for all twelve subjects and compared using their log-evidence. Fig. 3 shows the differences in log-evidences for each subject. For all but one subject, there was decisive evidence for the NMM over the MFM. The log-evidence at the group level (> 100), pooled over all subjects (given the data are conditionally independent over subjects) was similarly decisive. Although the relative log-evidence is quantitatively meaningful in its own right, one can also treat it as a log-odds ratio and use its distribution over subjects to compute a classical *p*-value (Stephan et al., 2010). In this instance, a one-sample *t*-test was extremely significant (*T* = 3.58, df = 11, *p* = 0.002). This means that we can reject the null hypothesis that the data are explained equally well by neural-mass and mean-field formulations of the same neuronal model.

**Fig. 4:**
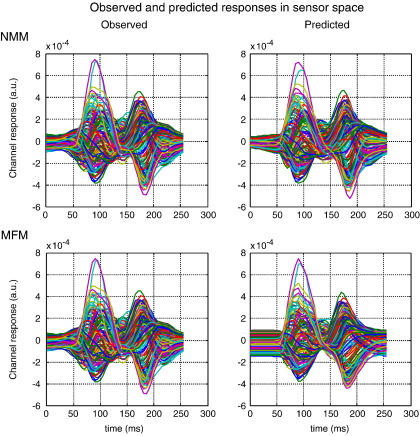
Upper panels: observed (left) and predicted (right) evoked responses over 128 channels and peristimulus time. Each coloured line corresponds to a different channel. These results are from the neural-mass DCM of the first subject from the MMN paradigm. Lower panels: the same but showing MFM predictions. The observed response is duplicated because it is adjusted for the confounding DC or constant term in our model (see Eq. (5)). This adjustment renders the observed data slightly different, depending on the model fit.

**Fig. 5:**
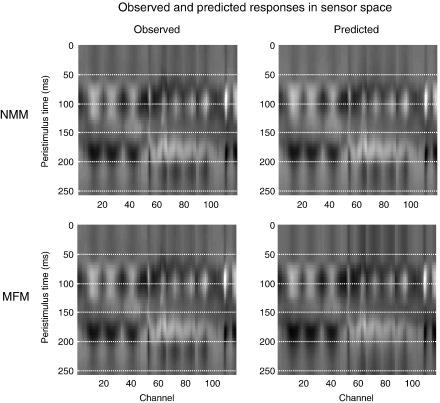
These are the same data as shown in Fig. 4 but in image format. Upper panels: observed (left) and predicted (right) evoked responses over 128 channels and peristimulus time (grey scale normalised to the maximum of each image). These results are from the NMM-DCM of the first subject from the MMN paradigm. Lower panels: the same but showing MFM predictions.

These results suggest the NMM is a better model for explaining evoked auditory responses. Note that the complexities of the NMM and MFM are the same; the MFM has more states but does not have more unknown parameters. This may seem counterintuitive because the dispersion in the MFM may appear to make it more complicated. However, the dispersion is a sufficient static of a density on hidden states and is not itself subject to random effects. This means, given the model parameters, it is a deterministic quantity and does not add to model complexity (i.e., it is specified by the same parameters as the neural-mass model). This is important because the results in Fig. 3 are remarkably consistent over subjects and cannot be explained by differences in model complexity. In short, the NMM provides a better prediction of the observed responses than the MFM, in this paradigm. Furthermore, the differences between the NMM and MFM predictions are fairly subtle (see Fig. 4). This suggests that the population variance is actually quite stable over peristimulus time, because the model selection clearly favours the predictions from the neural-mass model (Fig. 5).

### Simulations

We next performed some comparative evaluations and validations of DCM using neural-mass and mean-field models, using synthetic data based on the empirical results above. These are presented to show that the empirical model comparison above is sufficiently sensitive to disambiguate between neural-mass and mean-field variants of the same model. After generating data from a known model, we used model comparison to ask whether one can recover the correct model over its alternative. We integrated the NMM and MFM with known (true) model parameters derived from the real data above (the conditional means from a DCM of the grand average over subjects) and added random measurement noise with a standard deviation of 10% of the peak response in channel space. This was roughly the amplitude of noise in the real data—see Fig. 4. Finally, we used the synthetic data generated by both models to invert the neural-mass and mean-field DCMs. Table 2 lists the resulting log-evidences. Each column contains the log-evidences for each data set. The maximum values are found on the diagonal; i.e., the true model had the greatest evidence and the relative evidence for the correct model was ‘decisive’. These results confirm that these models can be disambiguated using DCM, under empirically realistic levels of noise.

### Somatosensory evoked potential paradigm

To explore the context sensitivity of these results, we analyzed data from a study of paired associative stimulation (PAS) (Litvak et al., 2007), which involves repetitive magnetic cortical stimulation timed to interact with median nerve stimulation-induced peripheral signals from the hand. The PAS paradigm has been shown to induce long-lasting changes in somatosensory evoked potentials (Wolters et al., 2005) as measured by single-channel recordings overlying somatosensory cortex. The SEP generators evoked by compound nerve stimulation have been studied extensively with both invasive and non-invasive methods in humans and in animal models (Allison et al., 1991). Litvak et al. (2007) characterised the topographical distribution of PAS-induced excitability changes as a function of the timing and composition of afferent somatosensory stimulation, with respect to a transcranial magnetic stimulation (TMS). The temporal response pattern of the SEP is composed of a P14 component generated subcortically and then an N20–P30 complex from the sensorimotor cortex, which is followed by a P25–N35 complex (Allison et al. 1991). The remainder of the SEP can be explained by a source originating from the hand representation in S1 (Litvak et al., 2007).

We chose these data as examples of fast sensory transients that might engage a more circumscribed network than the auditory stimuli in the MMN paradigm above. We anticipated that the density dynamics of neuronal populations that were stimulated electromagnetically, may disclose the effects of dispersion (see above). We analyzed the SEP data from eleven subjects following median nerve stimulation (i.e., in the absence of transcranial magnetic stimulation) as above. The network architecture was based on previous reports (Buchner et al., 1995; Ravazzani et al., 1995, Litvak et al., 2007): we modelled the somatosensory system with three sources, each comprising three neuronal populations. In this model (see Fig. 2, Litvak et al., 2007 and Marreiros et al. 2008) exogenous input was delivered to the brainstem source (BS), which accounts for early responses in the medulla. The input was a mixture of two parameterized bump functions with prior latencies based on known conduction delays (see Table 1). This region connects to two sources SI and SII in Brodmann area 3 (Marreiros et al., 2008). We inverted the resulting DCMs using the sensor data from 4 ms to 64 ms, following stimulation. We report the results form the first ten subjects because the DCM inversion failed to converge for the last subject.

### Empirical results

Fig. 3 (right) shows the log-evidence differences. In stark contrast to the MMN results, there was ‘decisive’ evidence in all but one subject for the MFM over the NMM. Moreover, the large group difference in log-evidence of (> 100) favours the MFM model; i.e., if we had to account for the data from all subjects with the same model, then the evidence for the MFM was decisive. The classical *p*-value was similarly significant (*T* = 2.19, df = 9, *p* = 0.028) but less significant than in the MMN analyses due to larger inter-subject variability. These results indicate that the MFM is, in this instance, a demonstrably better explanation for somatosensory evoked potentials. It is important to appreciate that exactly the same model was used for the MMN and SEP data, including the prior density on the free parameters (with the exception of the exogenous input). However, the results of model comparison are, as anticipated, completely the opposite and remarkably consistent over subjects for both paradigms.

**Fig. 6:**
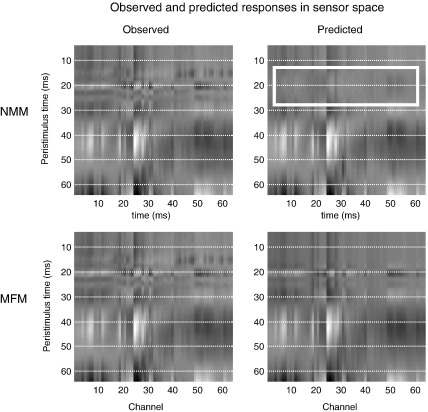
Observed and predicted responses from the SEP paradigm in the first subject. These images adopt the same format as Fig. 5 but showing responses over 64 channels and 4 ms to 64 ms of peristimulus time. The key thing to note here is the failure of the neural-mass DCM to model the early (N20) components of the SEP (white rectangle), relative to its mean-field homologue.

Anecdotally, the superior performance of the mean-field model seemed to be its ability to fit both the early N20–P30 complex and later waveform components of the SEP (although no model was able to fit the P14 components convincingly). This contrasts with the neural-mass model that was unable to reproduce the fast initial transients but was able to model the slower components that followed. The results from the first subject in channel space illustrate this difference nicely (Fig. 6); showing that the predictions of both models are almost indistinguishable after 30 ms. Phenomenologically, this means that the dispersion of neuronal states in the MFM confers a greater range on the time constants of population dynamics, which allows the MFM to reproduce fast, large-amplitude responses in, we presume, relatively circumscribed neuronal populations responding synchronously to extrinsic afferents.

### Simulations

To ensure the model comparison retained its sensitivity in this SEP setting, we again generated synthetic data using the conditional means of the parameters estimated from the empirical SEP data. We used an NMM and an MFM for generation and inversion and evaluated all combinations to ensure that model selection identified the correct model. For the integration of the forward models, we used the conditional means of parameters over subjects. We added random noise to these synthetic data, with a standard deviation that was 5% of the peak response in sensor space. We used the three-source model above (Litvak et al., 2007; Marreiros et al., 2008) to generate and model data. Table 3 presents the log-evidences for each of the four inversions. The highest evidences were obtained for the models that were used to generate the synthetic data: these correspond to the diagonal entries. Again, the results conform that model comparison can identify the correct model of these somatosensory responses, and do so decisively.

### A quantitative illustration of density dynamics

**Fig. 7:**
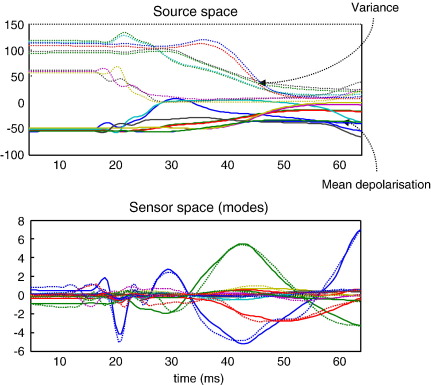
Standard DCM output for MFM of the SEP data. Upper panel: conditional estimates of the mean (solid lines) and covariance (dotted lines) of neuronal states, in source space (coloured lines correspond to different neuronal subpopulations). Lower panel: conditional estimates of responses, in sensor space (coloured lines correspond to different spatial modes; solid line: predicted; dotted line: observed).

Fig. 7 (upper left panel), shows the sufficient statistics of population activity for source in the first SEP subject. These are the *mean* and *covariance* of neuronal states, in source space. These are obtained by integrating the ensemble dynamics in Eq. (1), using the equations of motion in Eq. (3) and Fig. 1 and the conditional parameter estimates. Generally, when the mean depolarization increases, the covariance decreases, getting close to zero when the mean approaches its maximum. This is seen here at about 30 ms. This concentration of neuronal states augments the decay of mean depolarization (see Eq. (1)). Note that at around 20 ms the N20 is modelled by polyphasic dynamics in the mean depolarization that rests on a coupling with dispersion. These coupling and ensuing dynamics are the ones missing in the neural-mass model. In the lower panel, we see the conditional response estimate, in sensor space, in terms of the observed (dotted lines) and predicted (solid lines) time series for all modes. These results are representative of DCM prediction accuracy.

## Discussion

We have introduced a mean-field DCM for M/EEG data, which approximates the full density dynamics of population activity in neuronal sources of observed electromagnetic responses with a Gaussian density. This work was motivated by the observation that neural-mass models, which consider only the first moment of the density of each neuronal population, can be seen as a limiting case of mean-field models (Marreiros et al., 2009). The mean-field model used physiological plausible priors with the hope of creating a reasonably realistic conductance-based model (cf., Rodrigues et al., 2006). We have shown, using model inversion and simulations that one can disambiguate between MFM and NMM models (Tables 2 and 3) and found that the NMM was the best model for explaining the MMN data. In contrast, we found that the MFM was a better model of the SEP data (Fig. 3), in the vast majority of subjects and at the group level. We deliberately chose these distinct data sets in the hope of disclosing this dissociation between neural-mass and mean-field models.

This difference in performance between the two models on the two data sets lies in the difference between Eqs. (1) and (2). Our results suggest that the MFM captures the faster SEP population dynamics better than the NMM. This may be because the SEP paradigm evokes larger presynaptic inputs to small circumscribed neuronal populations as compared to the MMN and related cognitive paradigms. It is this input that induces changes in conductance which synchronise hidden states and cause a subsequent suppression of mean activity. It can be seen from Eq. (1) that changes in covariance depend on the derivative of flow. Eq. (4) shows that this depends on depolarization and conductance. In support of this, the MFM solutions for the SEP data do indeed show a reciprocal coupling between mean depolarization and variance (see Fig. 7). Having said this, the appropriateness of a model for any particular data or paradigm data cannot necessarily be deduced analytically. The aim of this paper was to show that questions about density dynamics of this sort can be answered using Bayesian model comparison. Future studies with NMM and MFM may provide heuristics about the relative utility of these models. In particular, it will be interesting to use MFMs when more complex dynamics are induced by extreme perturbations from steady-state dynamics (e.g., transcranial magnetic stimulation).

Most DCMs in the literature are deterministic; in that they allow for observation noise on the sensors but do not consider random fluctuations on hidden states. Here, the hidden states in mean-field DCMs are sufficient statistics of a density, which accommodates random fluctuations on neuronal states. This is important because it means we can model systems specified in terms of stochastic differential equations (cf., Eq. (3)) with ordinary differential equations (Eq. (1)) through the Fokker-Planck formalism. A potential application of this approach, beyond finessing prediction of EEG signals, could be in the context of EEG-fMRI fusion; where the second-order statistics of neuronal activity (cf., power) may be an important predictor of BOLD signals.

It is important to appreciate that the scheme described in this paper is not tied to any particular model of neuronal dynamics. The same comparisons presented above could be repeated easily, using any model of neuronal dynamics that are entailed by their equations of motion. Indeed, we anticipate that people will want to compare neural-mass and mean-field implementations of different models (see software note). The only constraint on these comparisons is that the equations of motion should be nonlinear in the neuronal states. This is because linear models preclude a coupling of first and second-order moments and render the behaviour of the neural-mass and mean-field formulation identical.

### Conclusion

We have shown that it is possible to implement a mean-field DCM, which considers the mean and variance of neuronal population activity. The modulation of second-order statistics may be a useful extension of DCM for evoked responses, as measured with magnetoencephalography and electroencephalography. Critically, the role of higher moments can be assessed empirically in a Bayesian model comparison framework. In this initial work, we conclude that, although conventional neural-mass models may be sufficient for modelling responses in conventional cognitive paradigms, it is easy to find evidence for coupling among the moments of neuronal ensemble densities in observed EEG data.

## Software note

Matlab demonstration and analysis routines referred to in this paper are available as academic freeware as part of the SPM software from http://www.fil.ion.ucl.ac.uk/spm.

## Figures and Tables

**Table 1:** Prior densities on the neuronal parameters.

Free parameters	
Extrinsic coupling parameters	$\begin{array}{cc} \gamma_{ij}^{E}=\{\begin{array}{cc} \frac{1}{2}e^{\theta } & \text{forward}\\ \frac{1}{4}e^{\theta } & \text{backward}\\ \frac{1}{4}e^{\theta } & \text{lateral} \end{array} & p(\theta )=N(0,1) \end{array}$
Intrinsic coupling parameters	$\begin{array}{ccc} \gamma_{ij}^{E}=e^{\theta }\left[\begin{array}{ccc} 0 & 0 & \frac{1}{2}\\ 0 & 0 & 1\\ \frac{1}{2} & 0 & 0 \end{array}\right] & \gamma_{ij}^{I}=e^{\theta }\left[\begin{array}{ccc} 0 & \frac{1}{4} & 0\\ 0 & 0 & 1\\ 0 & 1 & 0 \end{array}\right] & p(\theta )=N(0,\frac{1}{32}) \end{array}$
Capacitance	$\begin{array}{cc} C=8e^{\theta } & p(\theta )=N(0,\frac{1}{32}) \end{array}$
Time constants	$\begin{array}{ccc} \tau_{e}=\frac{1}{\kappa_{e}}=4e^{\theta }\text{ms} & \tau_{i}=\frac{1}{\kappa_{i}}=16\text{ms} & p(\theta )=N(0,\frac{1}{32}) \end{array}$
Diffusion tensor	$\begin{array}{cc} D(\omega )=e^{\theta }\left[\begin{array}{ccc} \frac{1}{8} & 0 & 0\\ 0 & 1 & 0\\ 0 & 0 & 1 \end{array}\right] & p(\theta )=N(0,\frac{1}{64}) \end{array}$
Conduction delays	$\begin{array}{cc} \Delta_{ij}=\{\begin{array}{cc} 2e^{\theta }\text{ms} & \text{intrinsic}\\ 16e^{\theta }\text{ms} & \text{extrinsic} \end{array} & p(\theta )=N(0,\frac{1}{128}) \end{array}$
Stimulus function parameters	$u\left(t\right)=\exp\left(-\frac{{\left(t-\tau_{u}e^{\theta \text{latency}}\right)}^{2}}{\tau_{u}e^{\theta \text{duration}}}\right):\{\begin{array}{c} \tau_{u}=128\text{ms:MMN}\\ \tau_{u}=16\&32\text{ms:SEP} \end{array}p\left(\theta_{i}\right)=N\left(0,\frac{1}{16}\right)$
Fixed parameters	
Potentials	$V_{L}=-70\mu \text{V }V_{E}=60\mu \text{V }V_{I}=-90{\mu \text{V}}_{\text{1}}V_{R}=-40\mu \text{V}$
Conductance	*g*_*L*_ = 1

**Table 2:** Log-evidences for neural-mass (NMM) and mean-field (MFM) models using synthetic data generated by a five-source MMN model (see Fig. 2) using NMM and MFM formulations. The diagonal values in bold show higher log-evidences for the true model.

Models	Synthetic data	
	NMM	MFM
NMM	**− 662.7**	− 952.9
MFM	− 844.2	**− 665.5**

**Table 3:** Log-evidences for neural-mass (NMM) and mean-field (MFM) models using synthetic data generated by a three-source SEP model (see Fig. 2) using NMM and MFM formulations. The diagonal values in bold show higher log-evidences for the true model.

Models	Synthetic data	
	NMM	MFM
NMM	**− 185.0**	**−** 175.8
MFM	**−** 369.6	**− 102.1**
